# Amputation of the Palatal Roots of Superior First Molars for the Prevention of Antral Abscesses

**Published:** 1902-11

**Authors:** Isidore Lett

**Affiliations:** Boston, Mass.


### AMPUTATION OF THE PALATAL ROOTS OF SUPERIOR FIRST MOLARS FOR THE PREVENTION OF ANTRAL ABSCESSES.[1]

BY ISIDORE LETT, D.D.S., BOSTON, MASS.

ONE of the disagreeable cases which dentists are called upon to treat surgically is a purulent secretion in the antrum of Highmore. These conditions are frequently met with, and whereas formerly most cases were sent to general surgeons, now it is the work for advanced dentists.

---

[1] Read before the American Academy of Dental Science, Boston, Mass., May 7, 1902.

You all being familiar with the anatomy of the maxillary bones and antrum, I will proceed to give details of a treatment for preventing an antral abscess, caused by mephitic gases from a putrescent pulp passing through the apical foramen of palatal roots of superior first and second molar teeth which perforate the wall of the antrum. There has been very little written on this subject, if any at all. It is not an uncommon occurrence in cleansing out the palatal root to be able to pass the probe through the apical foramen into the antrum. In such cases in which the pulp had been devitalized by the dentist, or from other causes, before putrefaction has caused the mephitic gases to pass into the antrum, I should certainly not amputate such a root, but simply treat it as any ordinary case, but when it is a case of an old putrescent pulp, an enlarged apical foramen, allowing a small broach to pass easily through it, I should recommend the amputation of such a root. The palatal roots of second molars have been found to perforate the wall of the antrum. Personally, I have not met with such a case. It is advisable, when we find on examination a putrescent pulp in a first or second molar, to probe very carefully into the palatal root, for if we were to pass the broach up carelessly, it may happen to be one of those cases previously described, and possibly force some septic matter directly into the antrum. I usually begin by opening the buccal roots and disinfecting them, then proceed on the palatal root by first opening the mouth of the canal with a rose bur (S. S. White's No. 5 to No. 8), then approaching the apex by frequently and carefully removing the deleterious matter with the broach and syringe. If the apical foramen is of normal size, I leave it alone, but, on the contrary, should the broach pass through, then I probe to ascertain if there is an opening through into the antrum.

I will now give you the results of three operations, two being successful, and one, being quite complicated, a failure. In the fall of 1897 a patient was sent to me to have a bridge for the superior right side. The two bicuspids and second and third molars had been extracted. The first molar was badly decayed and contained a putrescent pulp. After cleansing out the cavity and pulp-chamber I opened the buccal roots and then the palatal. The broach passed through the apical foramen into the antrum. On withdrawal of the broach I found a thick, creamy, purulent substance closely resembling pus. The amount was too small to pass judgment on. I opened the apical foramen so as to admit the broach with a little cotton twisted on the end. This had more of that substance, which had a decided disagreeable odor, and was probably pus. Having only this tooth to depend upon for the bridge, something desperate had to be done, to try and save the molar. Amputation of the root suggested itself to me, and I proceeded at once to perform the operation. As before stated, all the root-canals having been opened, I disinfected and filled the two buccal roots, gutta-percha being used for root-filling. The mouth of the palatal canal being previously enlarged, I cleansed, dried, and sealed it with a pellet of temporary stopping. With a large wheel bur I cut a circular groove around the interior of the root below the bifurcation, and followed this up with a small fissure bur in a right angle attachment, until the root was separated from the crown. Before removing the root cotton was packed into the fissure to prevent the blood from oozing into the crown cavity, also to act as a wall to allow the amalgam to be firmly packed and make a serviceable filling. The removal of the root was accomplished by making a vertical incision through the soft tissues from about the middle of the length of the root to the free margin of the gum. After gently forcing the tissues back, the alveolar process was removed with the engine, giving a good exposure. Then introducing a flat-bladed elevator into the fissure (the cotton having been removed), and pressing downward, the amputated end was forced out sufficiently to be grasped and removed with a pair of small forceps. The opening into the antrum was enlarged and a fifty per cent. solution of phenol-sodique was used to wash the antrum. I kept the treatment up for several weeks and then removed the packing and allowed the alveolus to fill with granulations from the floor of the antrum up. This is done by decreasing the size of the gauze dressing at each visit. Should the dressing be discontinued immediately after the last washing out of the antrum, the granulations will not be as firm and solid. I saw this case frequently for three months, then unfortunately, the patient being called away, I lost all trace of him. The tooth was apparently as solid as it ever had been.

Another case was as successfully treated as the previous one, with the exception that the antrum was syringed out only once and granulations allowed to close the sinus.

There is always some particular case which will not give us the desired satisfactory results. An operation, or experiment, should not be discouraged or condemned because of a failure of the first case. The failure may be due to some abnormal condition, such as malformation and fused roots, or a poor physical condition, anæmia, or the presence of some specific disease, congenital or acquired, such as scrofulosis or syphilis. Under those conditions I should advise the abandoning of the operation. A dentist should always take into consideration the physical and mental condition of a patient. If it is not satisfactory to him a course of treatment should be suggested either by him, or, preferably, by the patient's physician, to bring up the vitality. I had a very interesting and complicated case several months ago, with unsatisfactory results.

Mr. A., about twenty-eight years of age, presented himself in my office, complaining of severe pain in the superior right maxillary bone, and soreness over the first and third molar teeth. (The second molar had been extracted early in life, allowing the third molar to occupy its former place.) Periodontitis was evident in both molars. It was much more severe in the third molar. It had a large cement filling over a capped pulp. The pulp capping was unsuccessful. The crown resembled a bicuspid in shape and size. It was impossible to open the canals. I advised extraction, which was done. The roots were solidly fused together, having the appearance of a single thick root. The apex had been slightly broken down by caries. The next day the patient returned with no pain in the region of the third molar, but, as he expressed it, "the symptoms had moved from the extracted tooth to the other." This molar had been treated and filled some time before. There was very little of the walls remaining. I removed the large filling and opened up the pulp-chamber to the canals. The meso-buccal canal was normal. The disto-buccal canal could not be opened to the apex. There appeared to be a curve at almost right angles, about an eighth of an inch from the apex. The palatal canal was cleansed out and a probe passed into the antrum. I decided to amputate the palatal root. Should the disto-buccal root give trouble later on, I would open up the alveolar process and trim off the end of that root. With the use of adrenalin and a five per cent. solution of cocaine I separated the palatal root from the body of the tooth. This was a difficult operation. The root was fused as much as an eighth of an inch from the bifurcation. The pain was quite severe after the root had been detached, so I decided to allow the root to remain until the next day. The patient was discouraged on his return, and demanded the extraction of the tooth. It was impossible to persuade him to allow me to complete the operation. The tooth was extracted. The disto-buccal root was just as I had diagnosed it. About an eighth of an inch from the apex the root described almost a right angle. I treated the case for a week and discharged it.

An interesting case of caries of the superior maxillary bone, of eighteen years' standing, came under my care about six months ago, which I will mention here, as it touches slightly on the subject of the evening. The caries extended from almost the median line to just beyond the second bicuspid. The probe almost reached the palatal root of the first molar. The filling had been lost in this molar, and it contained a putrescent pulp. I opened the root-canals and found the palatal root had an enlarged apical formen and perforated the floor of the antrum. I advised the extraction of this tooth, fearing the carious bone would cause some unlooked-for results.

I have never attempted to treat an infected antrum by amputating the palatal root and using the opening for the introduction of a canula for drainage. The process being soft and cellular, the probabilities are the pus infiltrated through to such an extent that the removal of the suspected bone would leave the tooth with very little, if any, support.

Occasionally the root of a second bicuspid perforates the wall of the antrum. When putrefaction of the pulp takes place an abscess draining into the antrum is liable to result. In the endeavor to save the tooth I have seen the antrum treated through the tooth. These cases are rarely successful. For a time—weeks, months, or several years—the suppuration will stop, then suddenly and unexpectedly the old symptoms return, and when they do they are more difficult to treat, having becoming chronic.

In 1897 I operated on a case of caries of the right palate bones. The diagnostic signs and symptoms were identical with those which would indicate purulent secretion in the antrum. A youth, seventeen years of age, complained of pain in the region of the superior right maxillary bone, infra-orbital neuralgia, and a sense of fulness over the entire region. The teeth appeared to be in good condition. The first molar had a large cement filling. This was removed and a putrescent pulp found. After cleansing out the root-canals I found the two buccal roots normal, but the flexible probe passed through the apex of the palatal root and met with no obstruction for over an inch. My diagnosis was an abscess draining in the antrum. This root diverged abnormally from the buccal ones. For this reason, before giving my diagnosis to the patient, I again passed the probe through the apex and worked it in until I expected to touch the opposite wall of the antrum. Much to my surprise, the patient exclaimed, "Doctor, the instrument is in my nose." I next examined the roof of the mouth. There was no indication of any trouble here, with the exception of a very small white spot, smaller than the head of a pin, on the right side, between the median line and first molar. It appeared so insignificant I paid no attention to it. At this time I was passing a blunt probe over the roof of the mouth to locate any tenderness, which would help me from a diagnostic point of view. I pressed upon this white spot gently and the probe slipped through into the nose. The palatal root did not perforate the floor of the antrum but pointed towards the nasal cavity. I treated and filled the root-canals and crown. The following day I removed quite a quantity of the palatal and alveolar processes. This was done by making a deep incision from the median line opposite the second bicuspid to the second molar, and another incision from the median line to the cuspid, making a flap, which was dissected down, exposing the carious bone. This was all curetted out, leaving an opening about the size of a nickel between the nose and mouth. The flap was replaced, and, as it seemed to adapt itself so readily, no mechanical means were taken to retain it in position. The granulation filled in rapidly, with the exception of one point, which was kept open intentionally for drainage and syringing the floor of the nose. In three weeks the entire surface was healed, and in six months new bone filled up the space formerly occupied by the carious bone.

I should like to say a few words about hydrogen dioxide. It never should be used unless there is a large opening to allow it to escape easily. I have no doubt but what considerable damage is done by syringing the dioxide through the apex of a root into tissue, either bone or soft, for exploratory purposes, without having sufficient drainage. A good example of the folly of using it to asepticize the alveolus of a superior central incisor occurred to me in my first month of practice. The apex was large enough to allow the passage of a very fine broach. I used a small syringe to insert into the root-canal, and when the dioxide passed through the apex into the alveolar process the pain was excruciating and the lip began to swell. In less than five minutes the swelling was as large as a good-sized apple.

Fortunately Dr. James E. Garretson was in the next room, and I sent for him. The swelling was still growing larger, until it became half as large as her head. He immediately advised me to open up through the process and drain the parts, which I did. It stopped the pain, but the tumefaction did not disappear for nearly two weeks. Dr. Garretson could assign no cause why the dioxide should cause such an emphysema. It was an inoffensive-looking incisor. I have never used it in a root-canal since, unless there existed another means of drainage. Since then I have heard of many similar cases from other dentists, but no such extensive emphysema.

Just see what a complicated condition would have resulted had that been one of those palatal roots we are discussing, instead of an incisor. Some of the gas eliminated by the action of the hydrogen dioxide on organic matter would have passed into the antrum, possibly carrying some deleterious particles of matter along with it, causing an abscess; surely so if it were one of those anomalous conditions of the antrum, such as the obliteration of the opening into the middle meatus of the nose, caused by hypertrophy of the bones. So I repeat again, one should be very cautious about syringing dioxide in an antrum through a root-canal unless there is a source of drainage elsewhere.

